# Oral High-Dose Thiamine Improves the Symptoms of Chronic Cluster Headache

**DOI:** 10.1155/2018/3901619

**Published:** 2018-04-18

**Authors:** Costantini Antonio, Tiberi Massimo, Zarletti Gianpaolo, Pala Maria Immacolata, Trevi Erika

**Affiliations:** ^1^Università Cattolica di Roma, Largo Agostino Gemelli, Roma, Italy; ^2^Centro Polispecialistico Giovanni Paolo I, Viterbo, Italy; ^3^Department of Neurological Rehabilitation, The “Villa Immacolata” Clinic, Viterbo, Italy

## Abstract

Cluster headache is a rare painful primary disorder occurring in either episodic or chronic patterns. Several authors found that the hypothalamus, the brain region regulating endocrine function and autonomic system, is involved in the pathophysiology of cluster headache. Some authors have found in patients affected by this disease abnormality in glucose metabolism. Considering the role of thiamine in brain function, in energetic metabolism, and in pain modulation, we treated a patient affected by cluster headache with oral high-dose thiamine. We report a 41-year-old man suffering from primary chronic cluster headache since the age of 15 years. The patient began oral therapy with high-dose thiamine in December 2016. Oral thiamine supplementation led to a dramatic improvement of the symptoms. The therapy was effective in reversing all the symptoms of the disease. Our observation suggests that a thiamine deficiency due to enzymatic abnormalities or to dysfunction of the circulation of thiamine in the intracellular space could cause a neuronal selective impairment in the centers that are involved in this disease and could have an important role in the pathogenesis of the symptoms of cluster headache.

## 1. Introduction

Cluster headache (CH) is a primary headache, characterized by recurrent short-lasting attacks (15 to 180 minutes) of extremely severe unilateral periorbital pain together with ipsilateral autonomic signs (lacrimation, nasal congestion, ptosis, miosis, lid edema, and redness of the eye) [1]. The precise causative mechanisms are not yet known. Several authors found that the hypothalamus, the structure regulating endocrine function and autonomic system, is involved in the pathophysiology of CH [1]. There is no therapy for CH. However, there are some effective treatments for both the acute painful attacks as well as for the prophylactic treatments. The course of the disease is unpredictable. If the CH does not have periods of regression, it can be defined as chronic cluster headache (CCH) [2, 3].

Some authors found in CH patients abnormalities of cerebral glucose brain metabolism, hypometabolism in the cerebellopontine area, perigenual anterior cingulate cortex, and prefrontal and orbitofrontal cortex during bout and out of bout [4–6]. The same authors supposed that a decreased metabolism determines a deficient top-down modulation of antinociceptive circuits in CH patients.

Recently, other authors speculated that thiamine could have an important role in relieving migraine [7]. In our previous study on the treatment of extraintestinal symptoms of ulcerative colitis we described the case of a patient who had weekly episodes of migraine type, which completely regressed with high-dose thiamine [8]. The same treatment showed effectiveness in relieving pain in fibromyalgia [9].

Thiamine-dependent processes are critical in glucose metabolism, and recent studies implicate a role of these processes in oxidative stress, protein processing, peroxisomal function, and gene expression [9]. Primary thiamine deficiency is caused by inadequate intake of thiamine. Secondary thiamine deficiency is caused by increase demand, impaired absorption, or impaired metabolism. In alcoholics, many mechanisms contribute to thiamine deficiency. Thiamine deficiency causes Beri-Beri and Wernicke-Korsakoff syndrome [10, 11].

We have been treating several patients affected by different neurodegenerative diseases, sporadic or genetic, with high-dose thiamine [12, 13]. The favorable results in motor and nonmotor symptoms we obtained thus far led us to think that the symptoms of other neurological diseases could be caused by an intracellular, focal thiamine deficiency as well. This deficiency could be linked to a dysfunction of thiamine transport or to structural enzymatic abnormalities.

We hypothesized that abnormalities of brain energy metabolism in CH could be linked to selective neuronal thiamine deficiency in the centers that are involved in this disease. Additionally, we have decided to treat with high doses of thiamine a patient affected by CCH to clarify the potential effect of thiamine in the therapy of the disease.

Written informed consent was taken from the patient. The study did not require approval by Ethic Committee as per the local retrospective observations.

## 2. Case Presentation

Patient is male, 41 years old, and of 95 kg weight. First attack of right periorbital headache was at the age of 15 (in 1991), some days after a motorcycle accident.

This first attack lasted about one hour; it recurred every day but at different times of the day for about one week. The severe unilateral periorbital pain was always associated with agitation and ipsilateral autonomic signs (lacrimation, nasal congestion, ptosis, lid edema, and redness of the eye). These episodes lasted a week and did not occur for some years. About three years later, at the age of 18, headache attacks reappeared with a frequency of two or three per day for 15 days. Subsequently, these attacks occurred approximately every year/a year and a half, with two attacks per day over a two-week period. No prophylactic treatment currently used (verapamil, lithium carbonate, topiramate, valproic acid, gabapentin, and baclofen) has given benefits. No therapy including oxygen, octreotide, local anesthetics, dihydroergotamine, or indomethacin was useful for the treatment of acute attacks. From the age of 20, the patient began to use Sumatriptan (only drug that was shown to be effective) in a 6 mg subcutaneous injection that, in 10 to 20 minutes, was able to stop the single attack whose pain was intolerable. However, over time, the frequency of the headache attacks intensified, until the patient was 32 years old, when the frequency of headache attacks became daily (three to four per day) and these were still treated with Sumatriptan. No other drug had ever been effective in the prevention and the treatment of individual attacks. Rarely, the headache appeared in the left head area. This situation lasted for six years, until 2013, when, following the extraction of a tooth enclosed in the right maxillary bone, headache attacks disappeared for three years. In January 2016, clusters reappeared daily (4-5 attacks), always with the same characteristics and without any headache-free period until the end of December 2016, when the patient began the treatment we proposed. During the period September–December 2016, the patient also took 75 mg of prednisone daily, orally, without any benefit. The patient's diet was free, without alcoholics.

The CH diagnosis has been confirmed by all major centers for the treatment of headache in Italy (National Institute of Neurology Carlo Besta of Milan, S. Raffaele Hospital in Milan and in Rome). The diagnosis of CH was based on clinical history, physical examination, and Nuclear Magnetic Resonance (NMR) imaging. On our first examination, the patient's clinical picture corresponded to the classification of ICHD3.

Common biochemical and haematological investigations were normal, as well as electroencephalogram, brain magnetic resonance imaging, and neurological examination. Plasma thiamine level was 64 microg/L (normal value: 28–85).

We performed the cluster headache quality of life scale (CHQ) [14]. CHQ is a rating scale assessing how much the headache influences the patient's quality of life. It is composed of 28 items and its total score ranges from 28 to 140 points (28 = cluster headache does not influence the quality of life; 140 = cluster headache influences maximally the quality of life). In addition, the patient also did the Visual Analogic Scale (VAS) test, which measures how much the person is satisfied with his/her life. This test asks the patient to express a value between 0 (completely unsatisfied) and 100 (completely satisfied) concerning his/her overall quality of life. Before starting the therapy with thiamine, the CHQ score was 107 points, and the VAS score was 40 points (see Table 1).

We started thiamine treatment with an oral dose of 250 mg in the morning. Every 3 days the dose was increased by 250 mg up to the dose of 750 mg. This procedure of increasing dosage was followed because even low doses on normal individuals or on individuals who do not need vitamin supplements may show side effects such as tachycardia, anxiety, and difficulties in falling asleep [8]. The patient repeated the neurological exam CHQ of life scale one month after the beginning of the therapy (see Table 1). The patient had a progressive improvement of the symptoms. The headache episodes decreased in frequency until they disappeared completely within 10 days. Subsequently, as sometimes the patient reported short-term episodes described as “a weight on the head,” the dose was increased to one gram per day. This, however, caused the recurrence of a headache episode every morning at 4 o'clock, perfectly on time. The dose reduction to 750 mg/day caused the complete absence of headache attacks.

In March 2017, the patient had been invited to suspend the treatment to verify whether the headache attacks would reappear, but the patient refused to halt the high-dose thiamine therapy worrying that the pain and other symptoms could return. In May 2017, the patient accidentally forgot to bring the thiamine during a short vacation. Approximately 48 hours after the last dose of thiamine, he had a typical headache attack lasting about 60 minutes. The pain was strong but slightly attenuated and, therefore, tolerable compared to previous episodes. Later, the patient started a slimming diet and reduced the daily thiamine dose to 500 mg without any recurrence.

## 3. Discussion

Our patient affected by CCH has been treated with thiamine with benefit. Several years before, the patient was diagnosed with CH from many neurology centers of excellence in Italy. The clinical features, in particular headache characteristics, duration of the attacks (lasting more than 30 minutes before the high-dose thiamine therapy), ancillary symptoms, and the absence of improvement with indomethacin treatment (typical for another type of headache, named chronic paroxysmal migraine), confirm the diagnosis of CCH.

There was also a temporal relation between thiamine supplementation and improvement of headache, and there was a temporal link between the treatment suspension and the return of the attacks. The patient had a favorable response to thiamine. These data may suggest that any abnormalities in thiamine-dependent processes could be overcome by a diffusion-mediated transport at elevated thiamine concentrations. In the presence of thiamine deficiency, the response to therapy is considered diagnostic [15]. The response of neurological symptoms to thiamine supplementation in patients with normal concentrations of plasma thiamine could be explained if referred to a form of thiamine deficiency due to structural enzymatic abnormality or to dysfunctions of the transport or the circulation of thiamine in the intracellular space [16]. A high number of recent data showed that thiamine actions are not limited only to a coenzymatic role, but even its noncoenzymatic roles are relevant, particularly in neuroprotection and, then, in neurodegenerative diseases [10, 11]. Genetic or sporadic disorders of thiamine metabolism that lead to neurological diseases can be treated with high doses of thiamine [16, 17]. The exact mechanism of thiamine responsiveness in these patients remains unknown.

Oral high-dose thiamine was effective in reversing the symptoms of CH. However, pain (and related disorders) is not a symptom of classic thiamine deficiency. In neuronal circuits that control sensory inputs, a severe dysfunction of thiamine-dependent processes could produce these dysfunctional symptoms.

There is an increasing number of reports on effects of thiamine and of its derivatives, benfotiamine and thiamine monophosphate or pyrophosphate, in modulating pain process, both in animal models and in human studies; in these reports the treatment with thiamine improved the pain relief and supported this other noncoenzymatic role of thiamine [18–20].

These clinical and experimental observations allowed supposing that symptoms featuring CCH could derive from a focal thiamine deficiency that determines a neuronal selective impairment. The administration of large amount of oral vitamin B1 increases the intracellular passive transport of the thiamine and thus the symptoms decrease when thiamine-dependent processes are led back to physiologic levels [16, 17, 21]. This effect of thiamine in neurological long-lasting diseases by reversing clinical symptoms may be probably not directly related to the regulation of glucose metabolism and suggests a neuronal dysfunction rather than a neurodegeneration.

As we write this report, the patient maintains the same clinical conditions, without any side effects. We hope that a lifelong use of high doses of thiamine in individuals affected by CH can allow keeping the attacks under control and limiting the progression of the disease. In literature, there is no mention of thiamine-related adverse effects even at high doses and for very long periods of time [12, 22]. However, we had observed that, increasing the dose to reach a better result in a patient affected by ulcerative colitis with fatigue, mild tachycardia and insomnia appeared. By reducing the dose, tachycardia reversed within a few days [8]. When the dose of thiamine is excessive for the patient's necessity, the patient may experience agitation, insomnia, and the reappearance of the neurological symptoms previously reversed, as observed during treatment of neurodegenerative diseases including Parkinson's disease, Friedreich's ataxia, and dystonia (personal data not yet published). In the case of our patient treated for CH, the increase of thiamine doses also led to the reappearance of the neurological symptoms. The mechanism that may lead to such a manifestation of previously disappeared symptoms is unclear. Previous neurological conditions were restored by reducing the vitamin dose.

In conclusion, we think that our report represents an important contribution to the issue; nonetheless, further experience is necessary to confirm the present observation.

## 4. Learning Points

In literature, there is no mention that thiamine deficiency could play a role in the pathogenesis of cluster disease symptoms.

The abnormalities in thiamine-dependent processes could be overcome by a diffusion-mediated transport at supernormal thiamine concentrations.

Cluster headache appears to be responsive to high-dose thiamine. Cluster headache symptoms could be substantially reduced or even canceled with a simple, inexpensive, innocuous, quick, and highly effective therapy.

## Figures and Tables

**Table 1:** *Cluster headache quality of life scale (CHQ)*. The table indicates the frequency with which cluster headache influences the events indicated in the questions below. Linkert's scale (gives a numerical value to each answer): never = 1; occasionally = 2; sometimes = 3; often = 4; always = 5. The higher the score is, the more the headache influences negatively the patient's quality of life. The scale goes from 28 (the headache does not influence the patient's life) to 140 (it influences it maximally).

	Before	After
(1) Did it avoid you leaving the house?	4	1
(2) Did it avoid making plans due to unpredictability of CH e.g. holidays?	5	1
(3) Did you feel unable to complete duties at work?	4	1
(4) Did you have difficulty in getting involved in leisure activities e.g. cinema, theatre, etc?	4	1
(5) Did you avoid crowded and noisy places e.g., public transport, pubs, etc?	5	1
(6) Did you feel that the severity of cluster headache affected your daily activities?	5	1
(7) Have you been less involved in family affairs e.g. interactions with children, planning holidays?	4	1
(8) Have you been unable to socialize/spend time with friends and family?	4	1
(9) Have you been unable to achieve your daily goals and carry out routines and chores?	4	1
(10) Did you feel less respected by others?	3	1
(11) Did you have problem with close personal relationships?	3	1
(12) Did you feel you were burden for family and friends?	3	1
(13) Did you feel self-conscious and uncomfortable about your appearance after a cluster headache attack (e.g. swelling redness of eyes and facial sweating, etc)?	4	1
(14) Did you feel that others are dismissive of your cluster headache?	3	1
(15) Did you feel aggressive?	3	1
(16) Did you feel bad about yourself, lose self-confidence or feel worthless?	2	1
(17) Did you feel like harming yourself or suicidal?	2	1
(18) Have you been irritable, impatient or less tolerant?	3	1
(19) Have you been forgetful e.g., missed appointments?	4	1
(20) Have you been unable to take care of your appearance (e.g. take a bath, put make-up on, change clothe etc)?	3	1
(21) Did you feel isolated, lonely or vulnerable?	3	1
(22) Did you find your pain is unbearable if untreated?	5	1
(23) Did you dread that the headache would not go away?	5	1
(24) Did you feel lacking in energy and constantly tired?	4	1
(25) Did you feel sleepy, worn out or less able to concentrate due to nocturnal attacks of CH?	5	1
(26) Did you have problems concentrating e.g., reading paper, watching TV, etc.?	5	1
(27) Have you been unable to think clearly?	4	1
(28) Did you feel tense or anxious?	4	1
*Total score: …/140*	107	28

VAS: it measures how much the person is satisfied of her/his life (0 = completely unsatisfied; 100 = completely satisfied). The score of our patient was 40 before therapy and 100 after therapy with thiamine.
